# Sintering Temperature Induced Evolution of Microstructures and Enhanced Electrochemical Performances: Sol-Gel Derived LiFe(MoO_4_)_2_ Microcrystals as a Promising Anode Material for Lithium-Ion Batteries

**DOI:** 10.3389/fchem.2018.00492

**Published:** 2018-10-16

**Authors:** Li Wang, Yuanchuan He, Yanlin Mu, Mengjiao Liu, Yuanfu Chen, Yan Zhao, Xin Lai, Jian Bi, Daojiang Gao

**Affiliations:** ^1^College of Chemistry and Materials Science, Sichuan Normal University, Chengdu, China; ^2^School of Electronic Science and Engineering, University of Electronic Science and Technology of China, Chengdu, China

**Keywords:** LiFe(MoO_4_)_2_ microcrystals, anode material, sol-gel process, sintering temperature, electrochemical performance

## Abstract

A facile sol-gel process was used for synthesis of LiFe(MoO_4_)_2_ microcrystals. The effects of sintering temperature on the microstructures and electrochemical performances of the as-synthesized samples were systematically investigated through XRD, SEM and electrochemical performance characterization. When sintered at 650°C, the obtained LiFe(MoO_4_)_2_ microcrystals show regular shape and uniform size distribution with mean size of 1–2 μm. At the lower temperature (600°C), the obtained LiFe(MoO_4_)_2_ microcrystals possess relative inferior crystallinity, irregular morphology and vague grain boundary. At the higher temperatures (680 and 700°C), the obtained LiFe(MoO_4_)_2_ microcrystals are larger and thicker particles. The electrochemical results demonstrate that the optimized LiFe(MoO_4_)_2_ microcrystals (650°C) can deliver a high discharge specific capacity of 925 mAh g^−1^ even at a current rate of 1 C (1,050 mA g^−1^) after 500 cycles. Our work can provide a good guidance for the controllable synthesis of other transition metal NASICON-type electrode materials.

## Introduction

With the increase of environmental pollution and the rapid depletion of fossil fuels, a significant worldwide interest has been driven into the exploitation of clean and renewable energy devices (Gu et al., 2015; Mohanty et al., 2017; Mu et al., 2017; Wang T. Y. et al., 2017; Luo et al., 2018). As one of the most promising energy storage devices, lithium-ion batteries (LIBs) have been widely applied in many fields, such as portable electronic devices and electronic vehicles (Wu et al., 2016; Cai et al., 2017; Zhang Q. B. et al., 2018), which are attributed to their excellent features including high energy density, long lifespan, no memory effect and environmental benignity (Hassoun et al., 2014; Jiang et al., 2018; Zheng Z. M. et al., 2018). Nowadays, graphite is definitely as the common used anode material in the commercial LIBs. However, its relatively low theoretical capacity of 372 mAh g^−1^ falls short to meet the ever-growing requirement and hinders its further application (Wang et al., 2010, 2015; Zhao and Byon, 2013; Xiong et al., 2015; Hu et al., 2016; Wang H. E. et al., 2017; Li et al., 2018). Therefore, it is urgent to search alternative anode materials with high capacity and good cycling stability.

During the past decades, numerous efforts have been devoted to develop novel anode materials, which can be divided into two types: alloy anodes (Si, Ge and Sn) and conversion anodes (transition metal oxides, transition metal sulfides, transition metal phosphides and transition metal nitride and so on) according to their lithium storage mechanism (Lu et al., 2018). Among the above-mentioned anode materials, molybdenum-containing metal oxides have given rise to considerable attention due to multiple oxidation states, high capacity and high energy density (Sharma et al., 2004; Tao et al., 2011; Zhang L. et al., 2017).

Generally speaking, transition metal molybdates can be classified into single metal molybdates and binary metal molybdates (Zhang L. et al., 2017). Single metal molybdates, such as CoMoO_4_ (Yang et al., 2015), MnMoO_4_ (Guan et al., 2016), and NiMoO_4_ (Park et al., 2018) etc., can deliver the capacity of ~1,000 mAh g^−1^ when adopted as anode material. Therefore, these molybdates have been received extensive attention lately. Whereas, for the binary metal molybdates, there are relative seldom relevant available reports although they have multiple oxidation states and can offer much higher capacity. As a kind of typical binary metal molybdates, LiFe(MoO_4_)_2_, which belongs to a novel NASICON-type material with a structure of triclinic symmetry (space *P*-1), is constituted of the deformational LiO_6_ octahedron and separated FeO_6_ octahedron, and these octahedrons are connected by the MoO_4_ tetrahedron (Devi and Varadaraju, 2012; Chen et al., 2014). During the discharge process, 15 mol electrons can be transfered along with the reduction of Fe^3+^ to Fe^0^ and Mo^6+^ to Mo^0^, leading to the theoretical capacity as high to 1,050 mAh g^−1^. That is to say, LiFe(MoO_4_)_2_ is a very promising anode material, which may be beneficial to largescale energy storage applications in the future. However, how to accurately control the microstructures is the key role in the synthesis of LiFe(MoO_4_)_2_ electrode material, which restricts its further development. Up to date, the synthesis of LiFe(MoO_4_)_2_ via solid state method (Chen et al., 2014) and sol-gel method (Devi and Varadaraju, 2012) have been reported, and the lithium storage mechanism for LiFe(MoO_4_)_2_ anode is also expatiated. Unfortunately, the controllable of the microstructures is not deeply discussed. It is well-known that the sol-gel processing conditions have a significant influence on the microstructures, especially for the sintering temperature. Particularly, the sintering temperature plays an important role and has remarkable influences on the microstructures (including the crystallinity, morphology and grain size) and properties of material (Bahiraei et al., 2014; Xia et al., 2015; Dubey et al., 2017). Hence, it can be concluded that the electrode materials obtained at different sintering temperatures have various microstructures and electrochemical performances. As far as we know, there is no available report about the influences of sintering temperature on the microstructures and electrochemical performances of LiFe(MoO_4_)_2_ electrode materials up to now.

Based on the urgent and neglected aspects in the controllable synthesis for the NASICON-type binary metal molydbates, the present work is aimed at systematical investigation the influences of sintering temperature on the microstructures for sol-gel derived LiFe(MoO_4_)_2_ microcrystals. The obtained LiFe(MoO_4_)_2_ microcrystals at different temperatures have been carried out a series of electrochemical performances tests, and the LiFe(MoO_4_)_2_ microcrystals with superior electrochemical performance can be obtained just through precisely controlling the sintering temperature. Our work can provide a good guidance for the precise synthesis of other transition metal NASICON-type electrode materials.

## Experimental

### Synthesis

**Figure 1:**
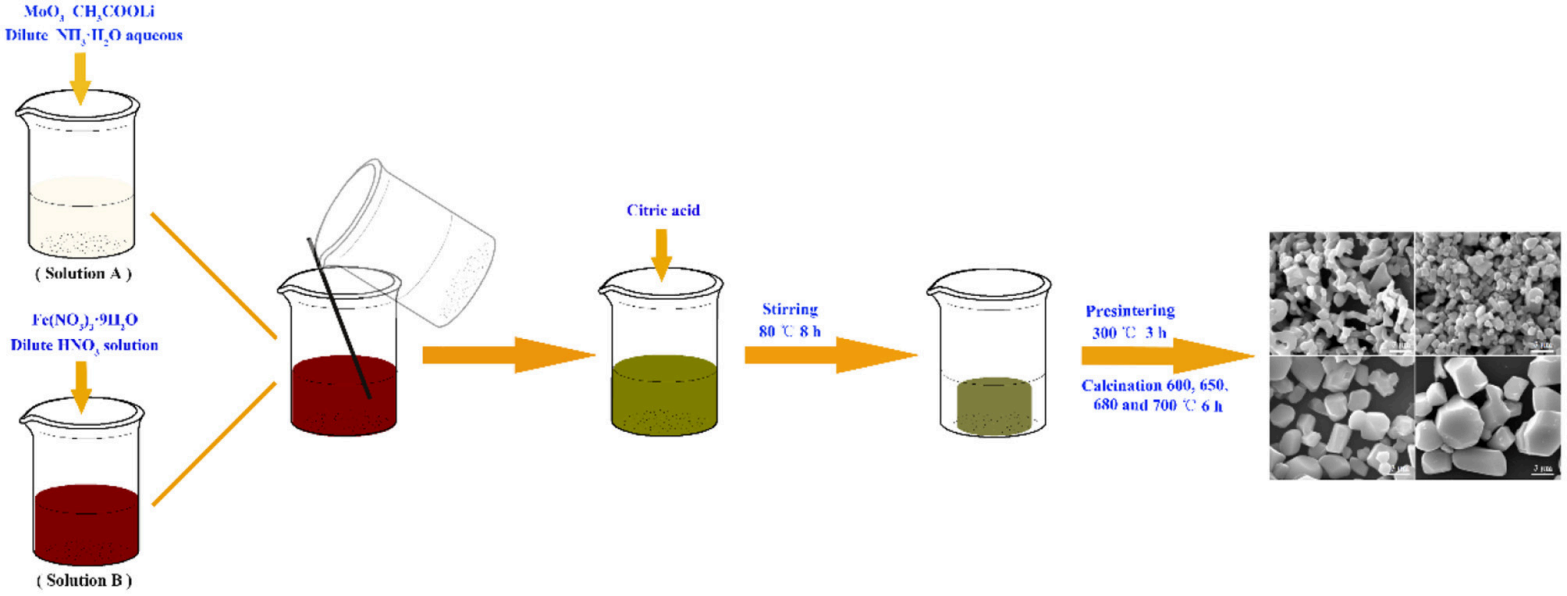
Schematic representation for the preparation of LiFe(MoO_4_)_2_ microcrystals.

All the reagents were of analytical grade and used without further purification. LiFe(MoO_4_)_2_ microcrystals were synthesized via a facile sol-gel method. A typical synthesis of LiFe(MoO_4_)_2_ microcrystals is depicted as follows: 1.7273 g of MoO_3_ and 0.3960 g of CH_3_COOLi were dissolved in 20 mL dilute NH_3_·H_2_O aqueous to form solution A, whereas 2.4240 g of Fe(NO_3_)_3_·9H_2_O was dissolved in 20 mL dilute HNO_3_ solution to form solution B, then the solution A and solution B were mixed together. Subsequently, 2.5217 g of citric acid was added into the above mixed solution under continuous stirring at 80°C to form gel. The obtained gel was further dried at 120°C in a vacuum oven over night. This dried gel was pre-sintering at 300°C for 3 h, and then the yellow precursors were calcined at 600, 650, 680, and 700°C for 6 h to obtain the final products, respectively. The obtained samples were denoted as LFM-600, LFM-650, LFM-680, and LFM-700, respectively. The synthetic procedures for LiFe(MoO_4_)_2_ microcrystals were illustrated in Figure 1.

### Characterization

The phase structure of the samples was characterized by X-ray diffraction (XRD, Rigaku miniflex) with Cu Kα radiation (λ = 0.15406 nm). The morphology was observed by scanning electron microscope (SEM, Quanta 250, FEI). X-ray photoelectron spectroscope (XPS) measurements were obtained on a Thermo Scientific Escalab 250Xi.

### Electrochemical performance measurements

The electrochemical performances of as-prepared products were evaluated using 2025-type coin cells, which were assembled in an argon-filled glove box with H_2_O and O_2_ contents of <0.5 ppm. The working electrode was prepared by mixing of 70 wt% LiFe(MoO_4_)_2_ powders, 20 wt% Super P Li (conducting additive) and 10 wt% polyvinylidene fluoride (PVDF, as binder) in the N-methyl pyrrolidone (NMP). The obtained slurry was coated onto copper foil and followed by drying at 100°C for 12 h in a vacuum oven. Lithium foil served as the counter electrode and 1 mol L^−1^ LiPF_6_ dissolved in ethylene carbonate/dimethyl carbonate (EC:DMC = 1:1, V/V) was used as electrolyte. Galvanostatic charge/discharge tests were carried out on a battery test system (LAND, CT2001A, China) between 0.01 and 3.0 V. The cyclic voltammetry (CV) and electrochemical impedance spectroscopy (EIS) measurements were performed on a CHI660E electrochemical workstation. The CV curves were investigated on a scanning rate of 0.1 mV s^−1^, while the EIS were measured at the frequency ranged from 0.01 Hz to 10 kHz.

## Results and discussion

**Figure 2:**
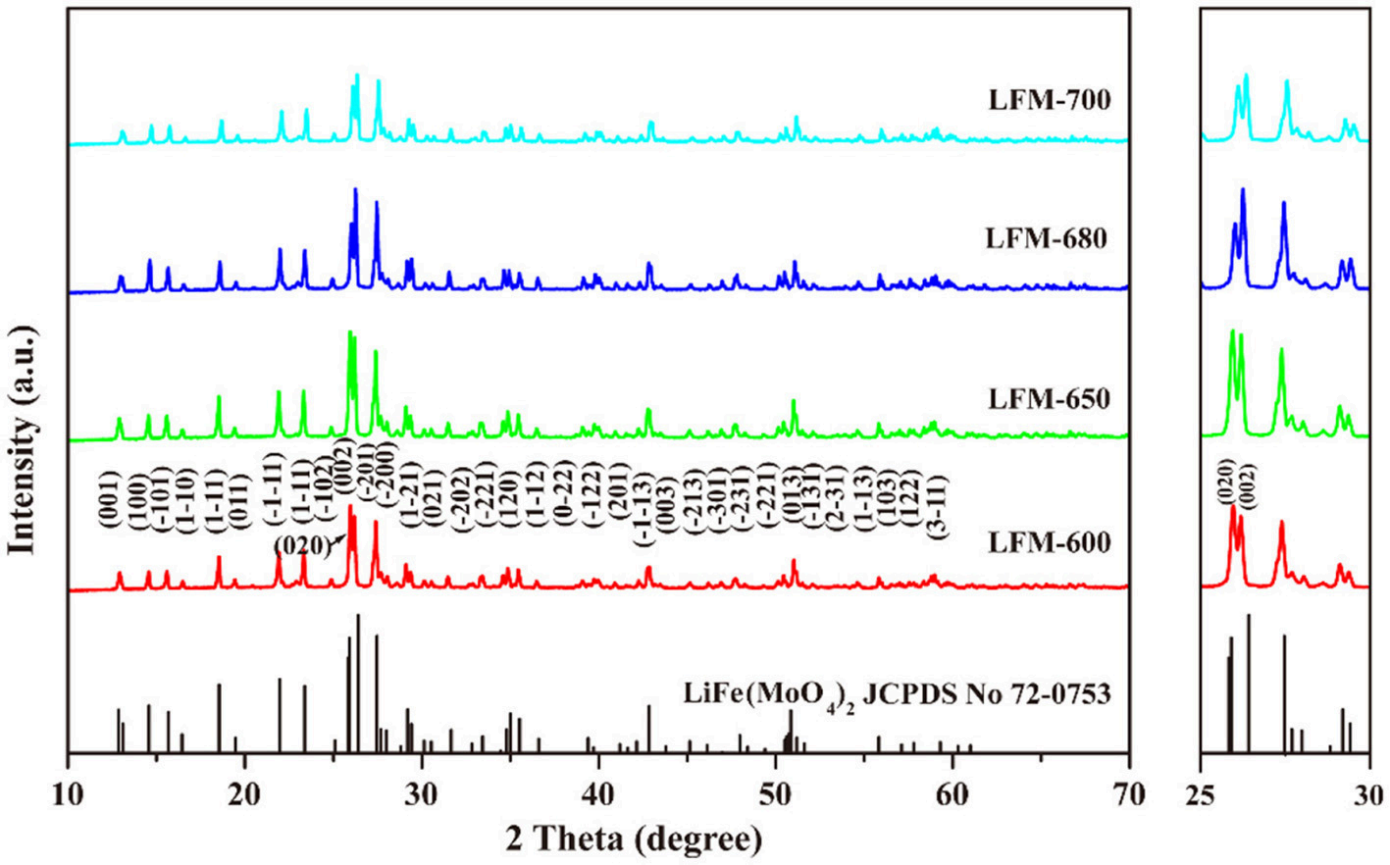
The XRD patterns of LFM-600, LFM-650, LFM-680, and LFM-700.

Figure 2 depicts the XRD patterns of LiFe(MoO_4_)_2_ microcrystals at different sintering temperatures. As can be seen, all the diffraction peaks of the four samples match well with the standard card of LiFe(MoO_4_)_2_ (JCPDS No. 72-0753), indicated that the pure phase products can be obtained at different sintering temperatures and the gained products belong to triclinic structure (space *P*-1). Careful observation, with the increase of sintering temperature, the relative intensity for (002) peak and (020) peak of the obtained LiFe(MoO_4_)_2_ samples exhibit slight discrepancy. When the sintering temperature is 600°C, the strongest diffraction peak of LFM-600 is (020) peak. While the sintering temperature increases to 650°C, the (002) peak and the (020) peak of LFM-650 have almost the same intensity. With further increasing sintering temperature, the strongest diffraction peaks of LFM-680 and LFM-700 turn into (002) peak. These phenomena indicate that there is an obvious orientation growth in the final LiFe(MoO_4_)_2_ microcrystals with the increasing sintering temperature, implying that they may possess various morphologies.

**Figure 3:**
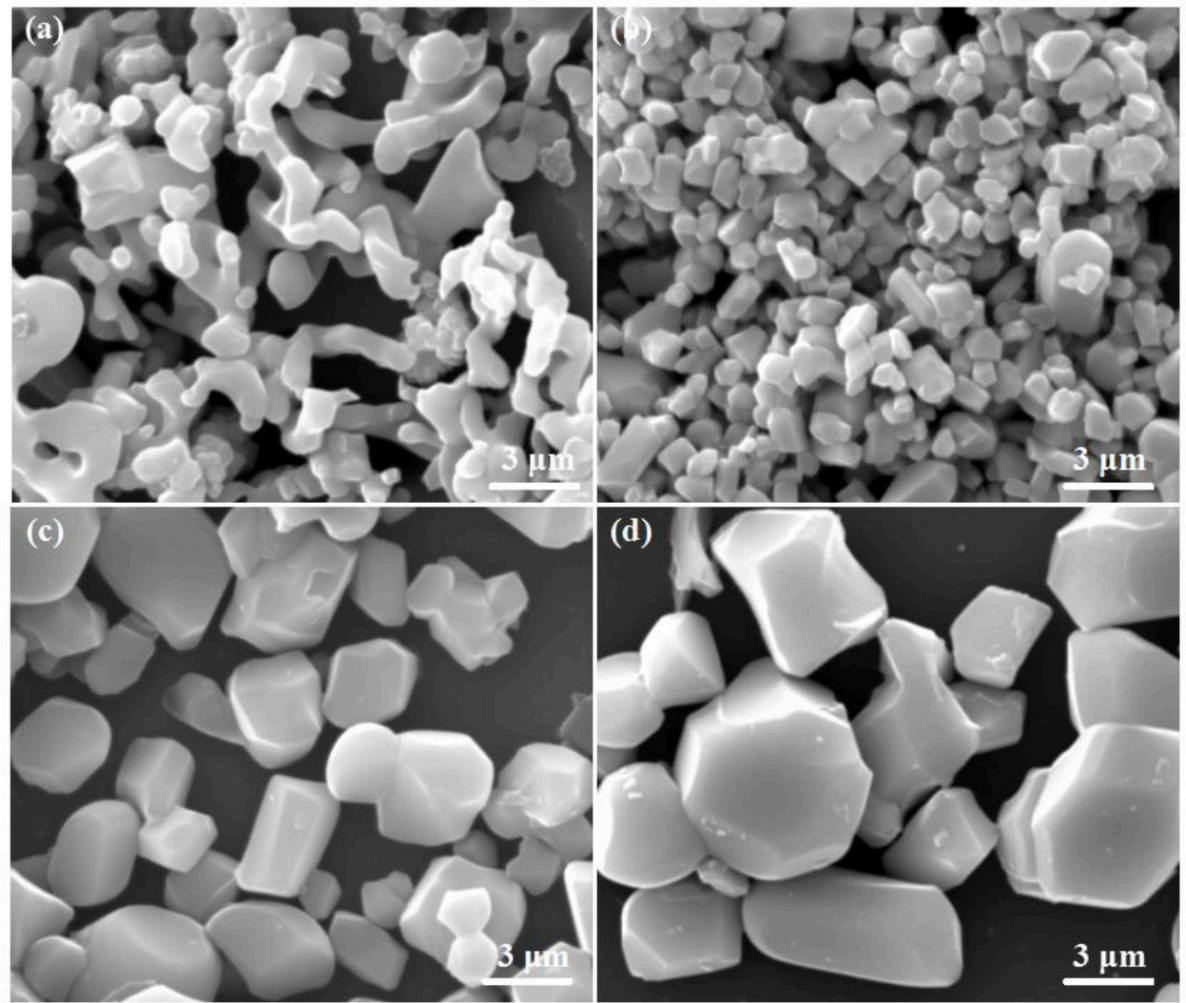
The SEM images of **(a)** LFM-600, **(b)** LFM-650, **(c)** LFM-680, **(d)** LFM-700.

The morphology and size of LiFe(MoO_4_)_2_ microcrystals under various sintering temperatures are shown in Figure 3. When the sintering temperature is 600°C, the obtained products reveal the irregular morphology and vague grain boundary, indicating that the as-obtained products possess relative poor crystallinity. When the temperature increases to 650°C, the obvious grain boundary can be observed, suggesting that the crystallinity of the products improved evidently. Careful observation, LFM-650 exhibits regular shape and uniform grain (mean size of 1–2 μm). This structure may favor the effective penetration of electrolyte. When the temperatures further increase to 680 and 700°C, the grain size of the products become larger and larger (mean size of 3–6 μm). The SEM results verify that the four samples possess various orientation growth direction, indicating that the sintering temperature really has a significant influence on the microstructures of the obtained LiFe(MoO_4_)_2_ microcrystals.

**Figure 4:**
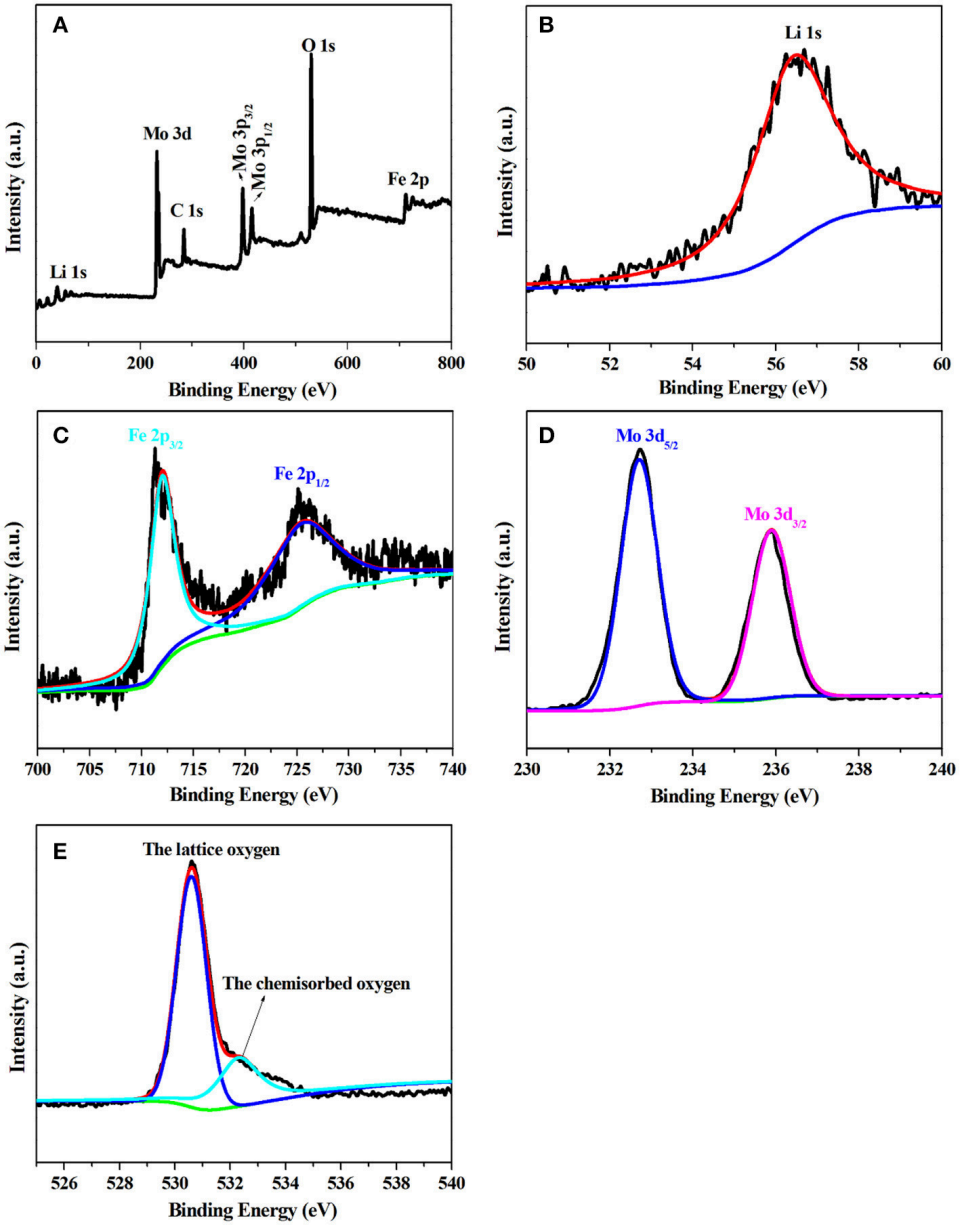
The XPS of LFM-650 **(A)** full XPS survey spectrum, **(B)** Li 1s, **(C)** Fe 2p, **(D)** Mo 3d, **(E)** O 1s.

To better investigate the chemical composition and valance state of LiFe(MoO_4_)_2_ microcrystals, LFM-650 is selected to perform XPS characterization, as presented in Figure 4. The survey spectrum (Figure 4A) shows the presence of Li, Fe, Mo, and O, along with thimbleful C from the reference electrode. It can be observed that the binding energy at 56.5 eV corresponds to the Li 1s, which is characteristic of Li^+^ (Figure 4B; Dedryvere et al., 2010). The two peaks with binding energies at 712.1 and 725.3 eV can be ascribed to Fe 2p_3/2_ and 2p_1/2_, representative of the existence of Fe^3+^ (Figure 4C; Zhang Y. Y. et al., 2017). In the high-resolution spectrum of Mo 3d (Figure 4D), two peaks with binding energies at 232.7 and 235.9 eV correspond to Mo 3d_5/2_ and Mo 3d_3/2_, which are assigned to the characteristic of Mo^6+^ (Yao et al., 2014). The O 1s XPS spectrum can be divided into two peaks, the binding energies at 530.6 and 532.3 eV are attributed to the lattice oxygen and chemisorbed oxygen, respectively (Gieu et al., 2017). The XPS results manifest that the chemical composition of the obtained LiFe(MoO_4_)_2_ microcrystals including Li^+^, Fe^3+^, Mo^6+^, and O^2−^, respectively.

**Figure 5:**
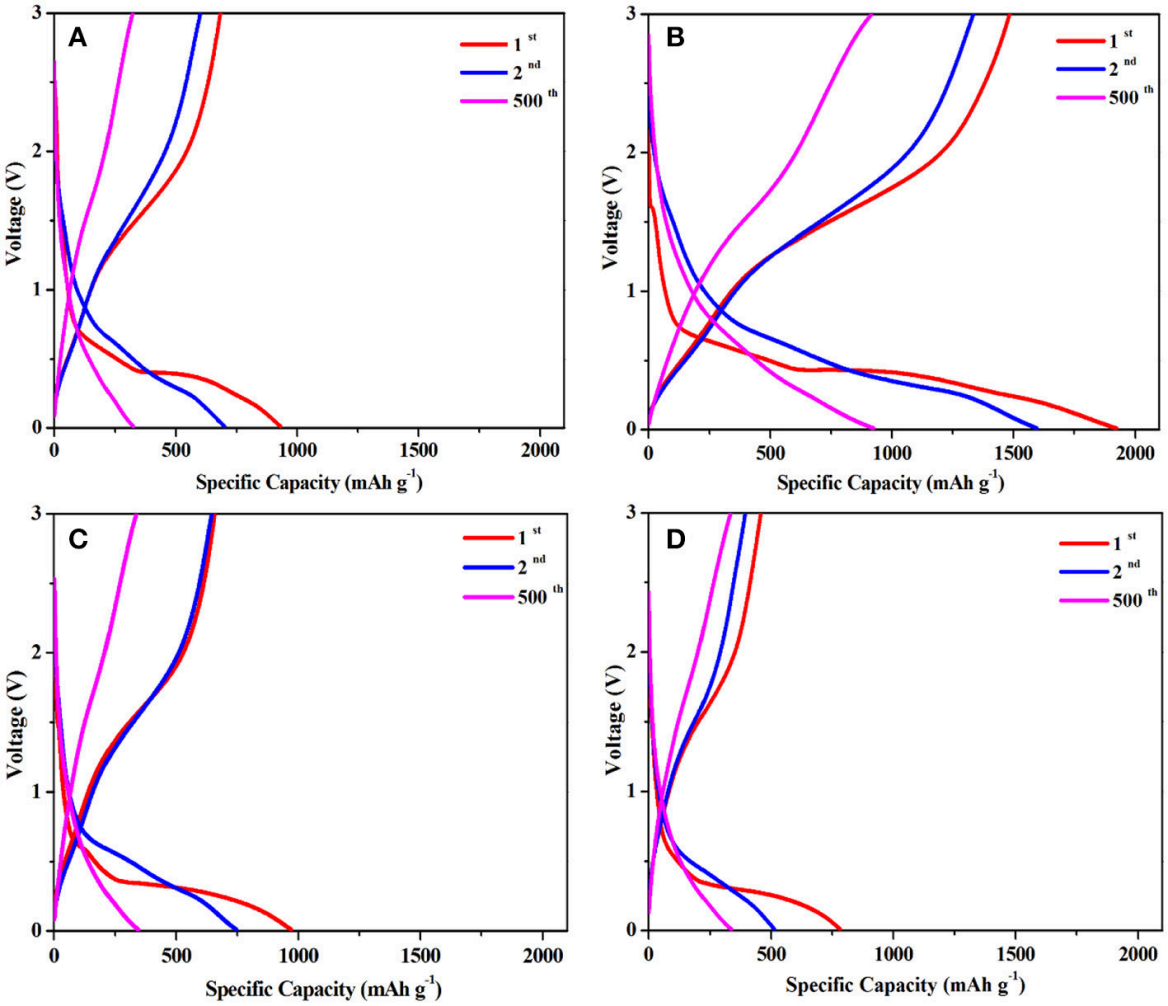
The discharge-charge curves of **(A)** LFM-600, **(B)** LFM-650, **(C)** LFM-680, **(D)** LFM-700.

Figure 5 exhibits the charge-discharge curves of LFM-600, LFM-650, LFM-680, and LFM-700 between 0.01 and 3.0 V at a current rate of 1 C (1,050 mA g^−1^) for the 1st, 2nd, and 500th cycles, respectively. As can be seen, the initial discharge specific capacity of LFM-650 reaches up to 1,923 mAh g^−1^, which is higher than those of LFM-600, LFM-680, and LFM-700, respectively. Although LFM-650 exhibits higher capacity, it also suffers from the low initial coulombic efficiency, which should be assigned to irreversible structural transformation of LiFe(MoO_4_)_2_ microcrystals and the formation of the solid electrolyte interface (SEI) film. It is worth noting that the discharge specific capacity of LFM-650 still can achieve 925 mAh g^−1^ after 500 cycles. Why the sample LFM-650 show such a high capacity? The answers can be gained by the XRD and SEM results. Comparison with the other samples, LFM-650 possesses optimal microstructure including good crystallinity, better uniformity and suitable grain size, which can enlarge the contact area between the active material and the electrolyte, bring much more active sites, profit the lithium ions and the electrons transportation, leading to superior electrochemical performance.

**Figure 6:**
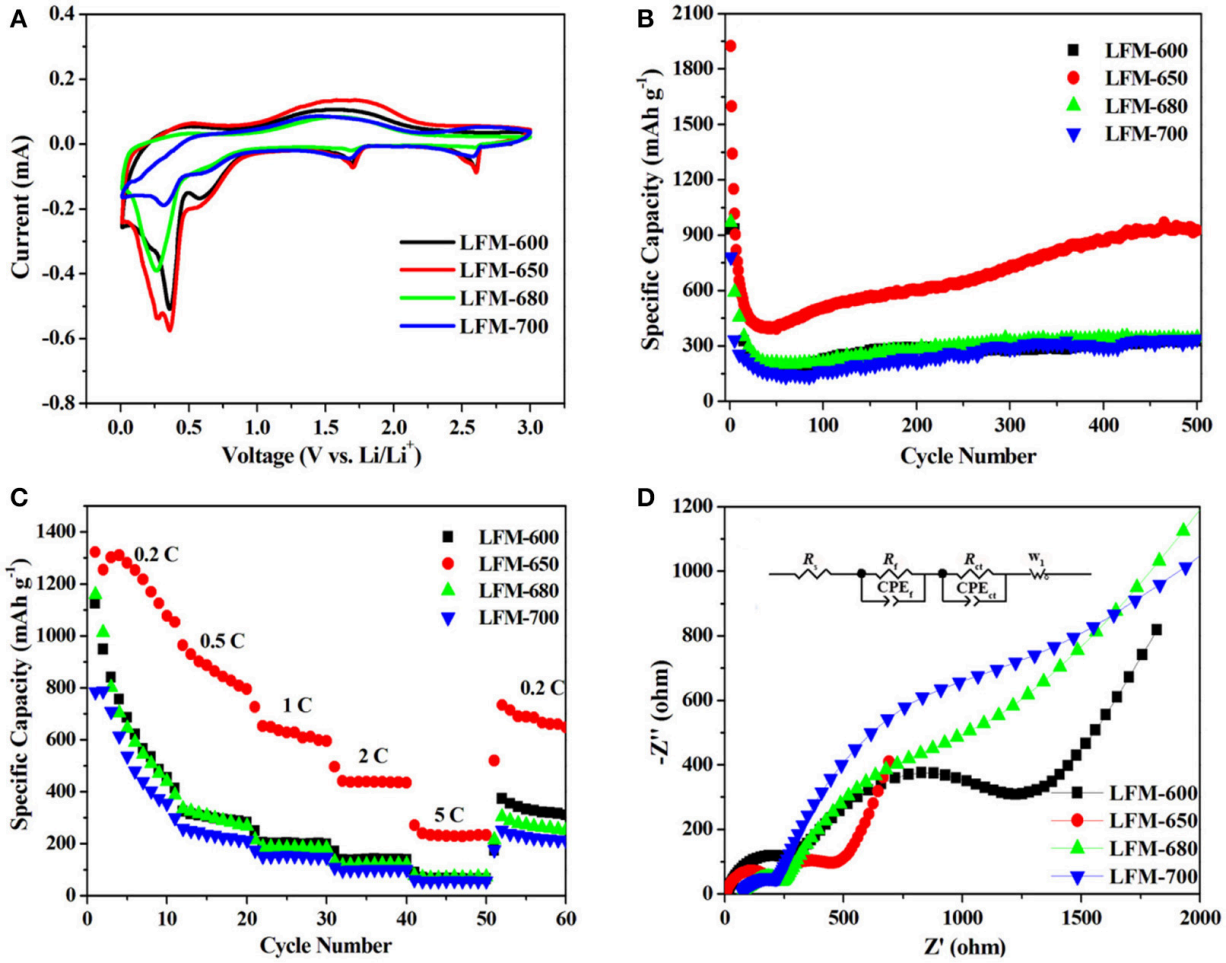
Electrochemical performances of the LiFe(MoO_4_)_2_ samples **(A)** the 1st CV curve at a scan rate of 0.1 mV s^−1^ between 0.01 and 3.0 V, **(B)** the cycling performance at a current rate of 1 C, **(C)** rate capability at different current rates, **(D)** Nyquist plots after the first discharge-charge cycle. The inset shows the equivalent circuit.

In order to evaluate the electrochemical performance of the four samples, various electrochemical measurements are carried out. Figure 6A presents cyclic voltammetry profiles of LFM-600, LFM-650, LFM-680, and LFM-700 for the 1st cycle in the voltage range from 0.01 to 3.0 V at a scan rate of 0.1 mV s^−1^, respectively. It can be observed that the four samples possess similar curves, which means that the redox process of the four samples during the charge-discharge process is identical. Careful observation, in the cathodic scan curve, the reduction peaks located at about 2.6 and 1.7 V are ascribed to the reduction of Fe^3+^ to Fe^2+^ and Mo^6+^ to Mo^4+^, respectively (Chen et al., 2014). The following reduction peaks at low voltage have a bit difference, but they also can be attributed to the reduction of Fe^2+^ to Fe^0^, Mo^4+^ to Mo^0^ and the formation of SEI film (Zhang et al., 2015). The reason that the four samples exhibit these differences can be speculated as follows: (i) Since the LFM-600 owns inferior crystallinity, only two obvious reduction peaks can be detected. In fact, there is a weak broad reduction peak locating at about 0.2 V. (ii) As for LFM-650, it possesses optimal microstructure including good crystallinity and suitable grain size, hence three legible reduction peaks can be easily observed. (iii) Both LFM-680 and LFM-700 show the relative larger grain size (diameter and height), which is adverse to the insertion/extraction of lithium ions and prolong the transport pathway of lithium ions, resulting in merely two reduction peaks appearance. In the anodic scan curve, the broad peak around 1.75 V is ascribed to the oxidation of Mo^0^ to Mo^6+^ and Fe^0^ to Fe^3+^. Interestingly, the above oxidation peak unexpectedly merges to a broad peak, hence the oxidation of Mo and Fe metals could not be distinguished. Moreover, the second and third scan curves for LFM-600, LFM-650, LFM-680, and LFM-700 are displayed in Figure S1. Clearly, the two curves are a bit different from the first curve, which can be assigned to the irreversible destruction of the LiFe(MoO_4_)_2_ structure and the formation of the SEI film (Gong et al., 2013; Wang et al., 2014). Further observation, the both curves of each sample exhibit good overlapping, indicating that the four samples have good reversibility, respectively.

Figure 6B displays the cycling performances of the four samples. Apparently, in the initial 40 cycles, the specific capacities begin to fade severely due to the formation of SEI film and the irreversible structural transformation (Xu et al., 2016; Wei et al., 2018). With the increase of cycling times, the specific capacities of the four samples gradually raise until to the stable value. These phenomena usually occur in transition metal oxide anode materials (Zhang et al., 2015; Guan et al., 2016), which can be attributed to the reversible growth of a polymeric gel-like film origination from kinetic activation process, and this can promote interfacial lithium storage. Therefore, the capacities gradually increase with extended cycling (Liu et al., 2018). It can be seen that LFM-650 can deliver a high discharge specific capacity of 925 mAh g^−1^ and own higher retention rate (88%, calculated based on the theoretical capacity of 1,050 mAh g^−1^) even at a current rate of 1 C after 500 cycles, which is far beyond LFM-600 (325 mAh g^−1^), LFM-680 (343 mAh g^−1^), and LFM-700 (337 mAh g^−1^), respectively. The higher capacity for LFM-650 can be ascribed to the good crystallinity and suitable grain size, which is beneficial for lithium ions transmission and repeating insertion/extraction. Comparatively speaking, LFM-600 has poor crystallinity, resulting in unsustainable longtime charge-discharge. Meanwhile, LFM-680 and LFM-700 possess larger grain size and this microstructure is disadvantageous to the insertion/extraction of lithium ions.

Figure 6C illustrates the rate capabilities of the four samples. Obviously, the specific capacities of LiFe(MoO_4_)_2_ microcrystals gradually fade with the increase of the current rate (from 0.2 to 5 C). Comparison with the four samples, the specific capacities of LFM-600, LFM-680, and LFM-700 are far below that of LFM-650 at different current rates. It can be observed clearly that the specific capacities of the above three samples fade severely, especially at high rates. Moreover, when the current rate comes back to 0.2 C, the specific capacities of LFM-650 can also reach 666 mAh g^−1^, which is much higher than those of other samples (LFM-600, LFM-680, and LFM-700), exhibiting superior rate capability. Fundamentally speaking, the distinction of the rate capability for LiFe(MoO_4_)_2_ microcrystals also may be related to the crystallinity and particle size.

To further understand why LFM-650 exhibits such superior electrochemical performance, electrochemical impedance spectroscopy (EIS) after the first discharge-charge cycle is carried out, as shown in Figure 6D. It is clearly observed that all the Nyquist plots of the four samples contain two parts, including two semicircles and slope line. The two semicircles at high and medium frequency region stand for the resistance of charge transfer in the electrolyte/electrode surface (*R*_ct_) and the resistance for the formation of SEI film in the electrode surface (*R*_f_), respectively. The slope line at low frequency reveals the Warburg impedance, which represents the diffusion of lithium ions in the bulk material (Sun et al., 2014; Li et al., 2015). The EIS data are fitted based on the equivalent circuit model (Wu et al., 2017), corresponding to the ohmic resistance (*R*_s_), the SEI film resistance (*R*_f_), dielectric relaxation capacitance (CPE_f_), the charge transfer resistance (*R*_ct_), double-layer capacitance (CPE_ct_) and Warburg impedance (W_1_), as presented in the inset of Figure 6D. The fitting results are tabulated in Table 1. It is obvious that the *R*_s_, *R*_f_, and *R*_ct_ for LFM-650 are much smaller compared with those of LFM-600, LFM-680, and LFM-700, demonstrating that LFM-650 possesses a more stable surface film and a faster charge transfer process, leading to the enhancement of lithium storage performance. More importantly, the notable increase in *R*_ct_ for LFM-600, LFM-680, and LFM-700 indicated that these electrode materials own higher kinetic barrier for lithium ions insertion/extraction, thus resulting in poor electrochemical performance (Zheng J. M. et al., 2018), just like what is displayed in Figures 6B,C.

**Table 1:** Fitted impedance parameters of LFM-600, LFM-650, LFM-680, and LFM-700.

**Samples**	***R*_s_ (Ω)**	***R*_f_ (Ω)**	***R*_ct_ (Ω)**
LFM-600	12.16	271.5	919.2
LFM-650	5.2	65.7	164.3
LFM-680	38.45	149.7	792.8
LFM-700	72.45	134.4	923.1

## Conclusion

In summary, LiFe(MoO_4_)_2_ microcrystals with pure triclinic structure have been successfully synthesized by a simple sol-gel method. The influence of sintering temperature on the microstructures and electrochemical performances was investigated in detail. The sample LFM-650 exhibits enhanced cycling stability and rate capability contrasted to the samples LFM-600, LFM-680, and LFM-700, which can deliver a high discharge specific capacity of 925 mAh g^−1^ even at a current rate of 1 C after 500 cycles. The superior lithium storage performance was attributed to the good crystallinity and uniformity as well as suitable grain size.

## Author contributions

LW designed and conducted the experiments. YH, YM, and ML helped the characterization and data analysis. LW wrote the paper. YC, YZ, XL, JB, and DG revised the paper.

### Conflict of interest statement

The authors declare that the research was conducted in the absence of any commercial or financial relationships that could be construed as a potential conflict of interest.
