# Supplementary material for: Sintering Temperature Induced Evolution of Microstructures and Enhanced Electrochemical Performances: Sol-Gel Derived LiFe(MoO4)2 Microcrystals as a Promising Anode Material for Lithium-Ion Batteries
Source: Front Chem. 2018 Oct 16;6:492. doi: 10.3389/fchem.2018.00492 (PMC6198042; doi:10.3389/fchem.2018.00492)
Supplement: Supplementary file 1 [file Data_Sheet_1.doc]

**Supplementary Material**

**Sintering temperature induced evolution of microstructures and enhanced electrochemical performances: sol-gel derived LiFe(MoO_4_)_2_ microcrystals as a promising anode material for lithium-ion batteries**

Li Wang^1^, Yuanchuan He^1^, Yanlin Mu^1^, Mengjiao Liu^1^, Yuanfu Chen^2^, Yan Zhao^1,2*^, Xin Lai^1^, Jian Bi^1^ and Daojiang Gao^1*^

^1^*College of Chemistry and Materials Science, Sichuan Normal University, Chengdu, 610068, P.R. China*

^2^*School of Electronic Science and Engineering, University of Electronic Science and Technology of China, Chengdu, 610054, P.R. China*

Corresponding authors: Yan Zhao and Daojiang Gao

E-mail addresses: zhaoyan@sicnu.edu.cn

daojianggao@sicnu.edu.cn; [daojianggao@126.com](mailto:daojianggao@126.com)


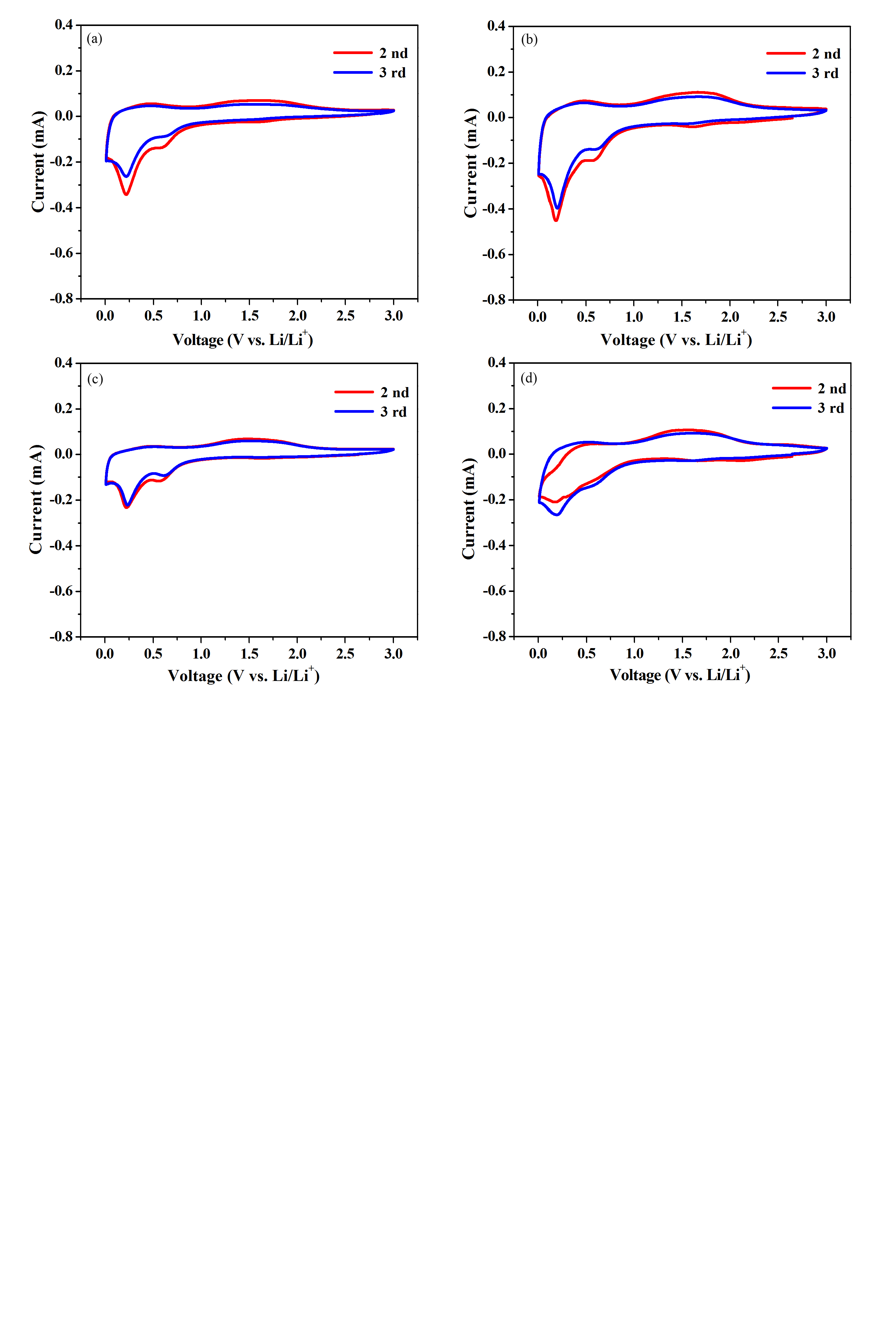


FIGURE S1 The 2nd and 3rd CV curves at a scan rate of 0.1 mV s^-1^ between 0.01 and 3.0 V (a)LFM-600, (b) LFM-650, (c)LFM-680, (d)LFM-700
